# Impact of bleeding during dual antiplatelet therapy in patients with coronary artery disease

**DOI:** 10.1038/s41598-020-78400-4

**Published:** 2020-12-07

**Authors:** Ying-Chang Tung, Lai-Chu See, Shu-Hao Chang, Jia-Rou Liu, Chi-Tai Kuo, Chi-Jen Chang

**Affiliations:** 1 Cardiovascular Department, Linkou Chang Gung Memorial Hospital, No. 5, Fusing St., Gueishan Dist., Taoyuan City, 33305 Taiwan, ROC; 2 College of Medicine, Chang Gung University, Taoyuan, Taiwan, ROC; 3 Department of Public Health, College of Medicine, Chang Gung University, Taoyuan, Taiwan, ROC; 4 Biostatistics Core Laboratory, Molecular Medicine Research Center, Chang Gung University, Taoyuan, Taiwan, ROC; 5 Division of Rheumatology, Allergy and Immunology, Department of Internal Medicine, Linkou Chang Gung Memorial Hospital, Taoyuan, Taiwan, ROC

**Keywords:** Cardiology, Medical research

## Abstract

This nationwide retrospective cohort study used the National Health Insurance Research Database of Taiwan to compare the impact of bleeding on clinical outcomes in patients with acute myocardial infarction (AMI) versus chronic coronary syndrome (CCS). Between July 2007 and December 2010, patients with AMI (n = 15,391) and CCS (n = 19,724) who received dual antiplatelet therapy after coronary stenting were identified from the database. AMI was associated with increased risks of MI (AMI vs. CCS: 0.38 vs. 0.16 per 100 patient-months; *p* < 0.01), all-cause death (0.49 vs. 0.32 per 100 patient-months; *p* < 0.01), and BARC type 3 bleeding (0.22 vs. 0.13 per 100 patient-months; *p* < 0.01) at 1 year compared with CCS, while the risk of BARC type 2 bleeding was marginally higher in the CCS patients than in the AMI patients (1.32 vs. 1.4 per 100 person-months; *p* = 0.06). Bleeding was an independent predictor of MI, stroke, and all-cause death in this East Asian population, regardless of the initial presentation. Among the patients with bleeding, AMI was associated with a higher risk of ischemic events at 1 year after bleeding compared with CCS (MI: 0.34 vs. 0.25 per 100 patient-months; *p* = 0.06; ischemic stroke: 0.22 vs. 0.13 per 100 patient-months; *p* = 0.02). The 1-year mortality after bleeding was comparable between the two groups after propensity score weighting. In conclusion, bleeding conferred an increased risk of adverse outcomes in East Asian patients with AMI and CCS.

## Introduction

Dual antiplatelet therapy (DAPT) with aspirin and a P2Y12 inhibitor has long been recommended as the cornerstone of pharmacologic treatment in patients with acute myocardial infarction (AMI) and chronic coronary syndrome (CCS) after they have received coronary stenting^1–3^. Advances in antiplatelet therapy have improved clinical outcomes of patients with coronary artery disease through the reduction of ischemic and thrombotic events, with the tradeoff of increased risk of bleeding. Depending on the definitions used, major bleeding rates within 30 days of contemporary percutaneous coronary intervention (PCI) range between 0.7 and 1.1% in elective procedures^4,5^, 0.6% and 4.7% in non-ST-elevation myocardial infarction (NSTEMI)^5–7^, and 0.9% and 8.9% in ST-elevation myocardial infarction (STEMI)^5,6,8,9^. Major bleeding complications have been well recognized as independently associated with short- and long-term mortality and major adverse cardiac events across the entire spectrum of CAD^4,10–12^.

East Asian populations exhibit relatively high platelet reactivity in response to clopidogrel treatment, but this relatively high on-treatment platelet reactivity does not appear to increase the risk of ischemic events, a phenomenon commonly referred to as the “East Asian paradox”^13,14^. Therefore, differences in patient characteristics and risk profiles may preclude the generalizability of findings regarding antiplatelet therapy from studies that mainly enroll patients from Western countries. Thus far, the incidence and prognostic impact of bleeding in East Asian populations have rarely been addressed. Data are also limited regarding the differences in the effects of bleeding on subsequent outcomes in patients with AMI versus CCS. In the present study, we used the National Health Insurance Research Database (NHIRD) of Taiwan to estimate the incidence rates of clinical events (bleeding, MI, stroke, and all-cause mortality) in patients with AMI and CCS who had received DAPT after coronary stenting. The effects of bleeding on subsequent clinical events (MI, stroke, and death) were compared between the two disease groups.

## Methods

### Data source

We used Taiwan’s NHIRD to conduct this nationwide retrospective cohort study. The National Health Insurance (NHI) Program is a compulsory health insurance program that currently covers approximately 99% of the population in Taiwan^15^. To protect patient privacy, the identification numbers of patients and names of health care providers and medical institutions in the NHIRD have been encrypted. The NHIRD contains claims data regarding the use of all medical facilities in Taiwan and provides patient-level data on diagnoses, demographic information, dates of outpatient visits and hospitalization, prescription drugs, and the use of medical procedures. Studies have validated the accuracy of the NHIRD with regard to the diagnoses of MI and stroke as well as mortality associated with these events^16–18^. The study design was in accordance of the ethical guidelines of the 1975 Declaration of Helsinki and was approved by the Institutional Review Board of Chang Gung Medical Foundation (No. 104-2932B). The requirement for informed consent was waived since the identification information in the NHIRD has been encrypted to ensure privacy.

### Study population

**Figure 1:**
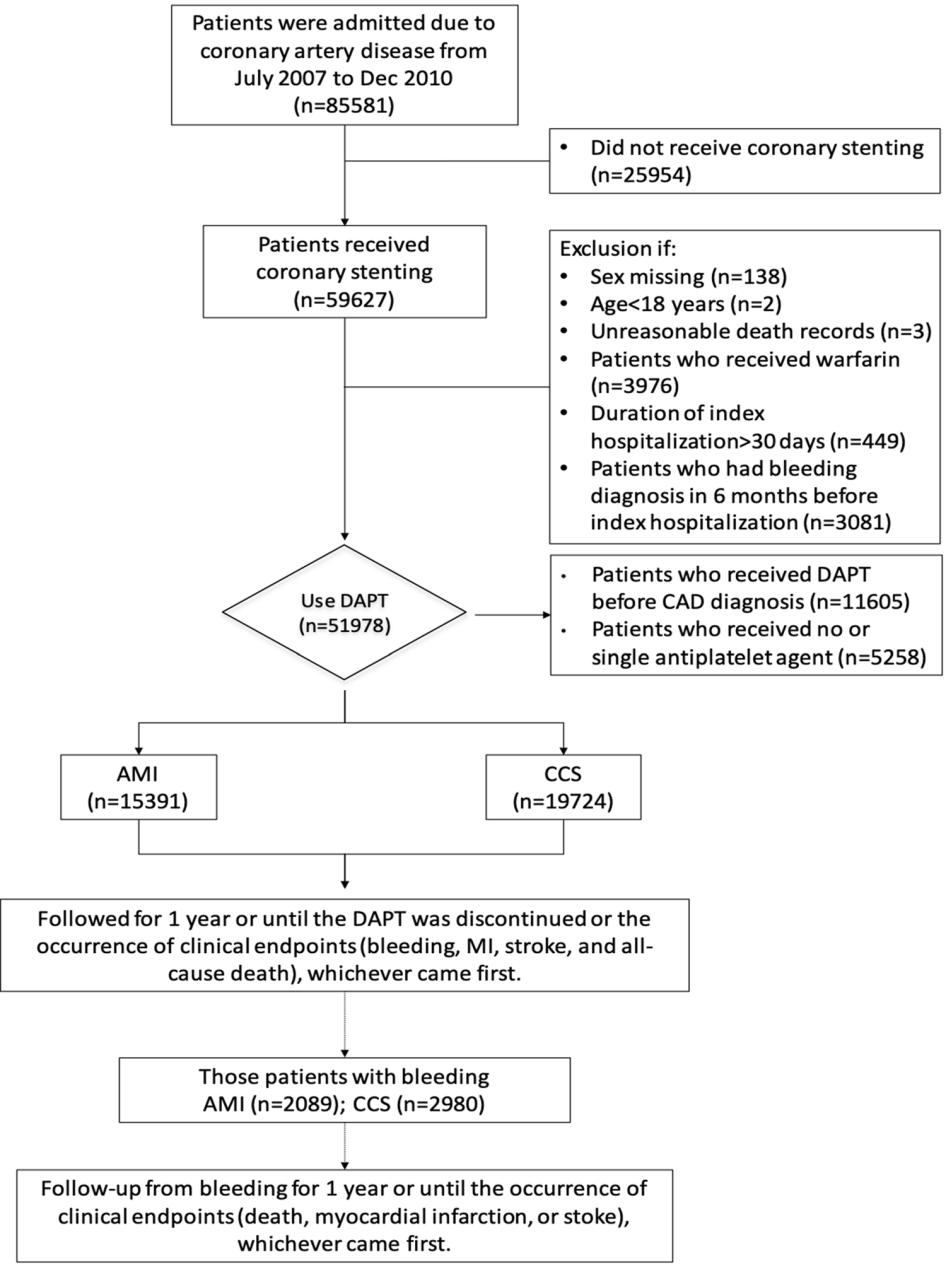
Patient enrollment. AMI, acute myocardial infarction; CAD, coronary artery disease; CCS, chronic coronary syndrome; DAPT, dual antiplatelet therapy.

Patients who were admitted for AMI or CCS and received DAPT after coronary stenting from July 1, 2007 to December 31, 2010 were eligible for this study. The exclusion criteria were the patients who did not undergo coronary stenting (n = 25,954) or did not receive DAPT after stenting (n = 5285), who received DAPT before the index hospitalization (n = 11,605) or required concomitant oral anticoagulation (n = 3976), who had prior bleeding (n = 3081), who were hospitalized for > 30 days (n = 449), who were younger than 18 years (n = 2), and those with missing data on sex (n = 138) or with unreasonable death records (n = 3). The study period predated the introduction of ticagrelor and prasugrel in Taiwan. Therefore, the DAPT regimen in this study was aspirin and clopidogrel. International Classification of Diseases, 9th Revision, Clinical Modification (ICD-9-CM) diagnosis codes were used for identification of AMI (410.x) and CCS (412.x, 413.x, and 414.x). Figure 1 is a flowchart of patient enrollment. A total of 35,115 patients who were admitted for coronary stenting and received DAPT for the first time were analyzed in this study.

### Study design

We used a dynamic cohort with two study groups, AMI (n = 15,391) and CCS (n = 19,724), to address two aims. First, to compare the incidence rates of clinical events during DAPT after PCI in patients with AMI versus CCS, we defined the date of PCI as the index date. All patients were followed for 1 year, until DAPT was discontinued, or the occurrence of clinical endpoints (bleeding, MI, stroke, and all-cause death), whichever came first. Another aim of this study was to examine the impact of bleeding on subsequent outcomes in patients with AMI versus CCS. Patients who had bleeding complications during DAPT were identified from the database and were followed from the date of bleeding for another 1 year or until the occurrence of clinical endpoints (MI, stroke, or all-cause death), whichever came first.

### Study outcomes

First, we compared the patients with AMI and patients with CCS for the clinical endpoints of bleeding, MI, stroke, and all-cause death during DAPT after coronary stenting. Bleeding endpoints were defined as Bleeding Academic Research Consortium (BARC) types 2, 3, and 5 bleeding^19^. BARC type 3 bleeding was defined as bleeding that requires blood transfusion or intravenous vasoactive agents, cardiac tamponade, intracranial hemorrhage, or intraocular bleeding. BARC type 5 bleeding was defined as bleeding being the principal diagnosis of admission with mortality within 7 days. BARC type 2 bleeding was defined as bleeding that requires medical intervention or evaluation but does not fit the criteria for type 3 or type 5 bleeding. The bleeding events were further categorized based on the sites of bleeding, including gastrointestinal, genitourologic, neurologic, airway, and other or nonspecific bleeding. The clinical endpoints of bleeding, MI, stroke, and all-cause death were calculated as independent events (i.e., the calculation of each of these events was independent from the others), whereas the subtypes of stroke (ischemic and hemorrhagic) and bleeding (BARC types 2, 3 and 5) were calculated as first events (i.e., only the first occurring subtype of stroke or bleeding was calculated). The impacts of bleeding on subsequent MI, stroke, and all-cause death were compared between the two disease groups.

### Statistical analysis

Propensity score weighting was used to ensure a balance between the AMI and CCS groups^20,21^. An absolute standardized mean difference of less than 0.1 was considered negligible between the two groups^22^. Comparisons of bleeding, MI, stroke, and death between patients with AMI and CCS were analyzed using the Cox proportional hazards model. Hazard ratios (HRs) and 95% confidence intervals (CIs) were obtained for each event using the CCS group as the referent group. The independent association between any bleeding (BARC type 2, 3 or 5) and MI, stroke, and death was examined using bleeding as a time-dependent covariate in the Cox proportional hazards model. Because some clinical events occurred more than once during follow-up, we used the Prentice, Williams, and Peterson model (PWP) model to account for recurrent events in multivariate survival data^23^. To evaluate the impact of bleeding during DAPT on subsequent MI, stroke, and death, we further identified patients with bleeding complications in the AMI and CCS groups, and performed outcome analysis using propensity score weighting and the Cox proportional hazards model. A *p* value of < 0.05 was considered to indicate statistical significance. All statistical analyses were performed using SAS version 9.4 (SAS Institute Inc., Cary, NC).

## Results

### Patient characteristics

Table 1 lists the baseline characteristics of the patients with AMI and CCS. Before propensity score weighting, the AMI patients were younger, more often male, with a higher rate of hyperlipidemia, more frequently prescribed angiotensin-converting enzyme inhibitors and statins, more frequently treated with unfractionated heparin, enoxaparin, glycoprotein IIb/IIIa inhibitors during hospitalization, and required periprocedural implantation of intra-aortic balloon pump. In comparison, the CCS patients were older; had higher rates of underlying diseases including diabetes, hypertension, chronic kidney disease, end-stage renal disease, chronic obstructive pulmonary disease, chronic liver disease, previous stroke, and anemia; and were more commonly prescribed angiotensin receptor blockers. There was no significant difference between the two groups with regard to the rate of thrombocytopenia and the use of proton-pump inhibitors, H2 blockers, steroids, and nonsteroidal anti-inflammatory drugs. The duration of DAPT was 297.5 days in the AMI group and 306.8 days in the CCS group. After propensity score weighting, there was no significant difference between the two study groups in terms of demographics, comorbidities, medications, inhospital management, and the duration of DAPT.

**Table 1:** Demographics and clinical characteristics of patients with AMI and CCS who had received DAPT after coronary stenting.

	Before propensity score weighting			After propensity score weighting		
	AMI (n = 15,391)	CCS (n = 19,724)	SMD	AMI (n = 14,840.13)	CCS (n = 19,534.12)	SMD
	n (%)	n (%)		n (%)	n (%)	
**Age, years**	62.57 (13.49)	65.53 (11.64)	− 0.235	63.57 (13.11)	64.71 (12.17)	− 0.091
≤ 75	12,216 (79.37%)	15,284 (77.49%)	− 0.046	11,533.24 (77.72%)	15,246.51 (78.05%)	0.008
> 75	3175 (20.63%)	4440 (22.51%)		3306.89 (22.28%)	4287.61 (21.95%)	
**Sex**			0.181			0.020
Female	3053 (19.84%)	5419 (27.47%)		3470.18 (23.38%)	4731.77 (24.22%)	
Male	12,338 (80.16%)	14,305 (72.53%)		11,369.95 (76.62%)	14,802.35 (75.78%)	
**Comorbidities**						
Diabetes mellitus	6290 (40.87%)	9463 (47.98%)	− 0.143	6714.59 (45.25%)	8763.32 (44.86%)	0.008
Hypertension	11,017 (71.58%)	16,725 (84.8%)	− 0.324	11,683.69 (78.73%)	15,499.73 (79.35%)	− 0.015
Atrial fibrillation	534 (3.47%)	1032 (5.23%)	− 0.087	651.40 (4.39%)	870.30 (4.46%)	− 0.003
Congestive heart failure	3462 (22.49%)	4359 (22.1%)	0.01	3350.88 (22.58%)	4362.76 (22.33%)	0.006
Chronic kidney disease	2101 (13.65%)	3644 (18.47%)	− 0.132	2461.30 (16.59%)	3213.61 (16.45%)	0.004
Chronic obstructive pulmonary disease	2317 (15.05%)	3961 (20.08%)	− 0.132	2583.77 (17.41%)	3490.28 (17.87%)	− 0.012
Chronic liver disease	1565 (10.17%)	2814 (14.27%)	− 0.125	1803.22 (12.15%)	2428.64 (12.43%)	− 0.009
Previous stroke	1208 (7.85%)	2139 (10.84%)	− 0.103	1441.40 (9.71%)	1861.74 (9.53%)	0.006
Hyperlipidemia	12,478 (81.07%)	15,104 (76.58%)	0.11	11,663.27 (78.59%)	15,344.93 (78.55%)	0.001
End stage renal disease	544 (3.53%)	1236 (6.27%)	− 0.127	751.60 (5.06%)	996.66 (5.1%)	− 0.002
Anemia	976 (6.34%)	1780 (9.02%)	− 0.101	1160.03 (7.82%)	1544.39 (7.91%)	− 0.003
Thrombocytopenia	35 (0.23%)	79 (0.4%)	− 0.031	34.82 (0.23%)	62.17 (0.32%)	− 0.016
**Medications**						
ACE inhibitor	12,274 (79.75%)	10,077 (51.09%)	0.632	9639.79 (64.96%)	12,427.27 (63.62%)	0.028
ARB	8400 (54.58%)	11,871 (60.19%)	− 0.114	8573.42 (57.77%)	11,248.13 (57.58%)	0.004
Beta-blocker	12,921 (83.95%)	16,015 (81.2%)	0.073	12,269.00 (82.67%)	16,128.31 (82.56%)	0.003
Statin	12,160 (79.01%)	14,124 (71.61%)	0.172	11,184.26 (75.36%)	14,621.62 (74.85%)	0.012
PPI	2995 (19.46%)	4382 (22.22%)	− 0.068	3128.26 (21.08%)	4051.41 (20.74%)	0.008
H2 blocker	1947 (12.65%)	2618 (13.27%)	− 0.019	1880.15 (12.67%)	2495.47 (12.77%)	− 0.003
Steroid	623 (4.05%)	912 (4.62%)	− 0.028	634.58 (4.28%)	843.39 (4.32%)	− 0.002
NSAID	2148 (13.96%)	2832 (14.36%)	− 0.012	2050.05 (13.81%)	2710.02 (13.87%)	− 0.002
**Inhospital management**						
Unfractionated heparin	12,753 (82.86%)	13,109 (66.46%)	0.384	11,166.28 (75.24%)	14,436.46 (73.9%)	0.031
Enoxaparin	5063 (32.9%)	5322 (26.98%)	0.129	4462.38 (30.07%)	5800.74 (29.7%)	0.008
GP IIb/IIIa inhibitor	4196 (27.26%)	649 (3.29%)	0.707	2128.62 (14.34%)	2689.98 (13.77%)	0.017
IABP	1503 (9.77%)	229 (1.16%)	0.386	763.29 (5.14%)	1006.55 (5.15%)	< 0.001
**Duration of DAPT**	297.54 (99.46)	306.77 (83.17)	− 0.101	302.95 (89.22)	302.53 (90.55)	0.005

*ACE* angiotensin-converting enzyme, *AMI* acute myocardial infarction, *ARB* angiotensin II receptor blocker, *CCS* chronic coronary syndrome, *DAPT* dual antiplatelet therapy, *GP* glycoprotein, *IABP* intra-aortic balloon pump, *NSAID* nonsteroidal anti-inflammatory drug, *PPI* proton-pump inhibitor, *SMD* standardized mean difference.

### One-year outcomes of AMI versus CCS

**Figure 2:**
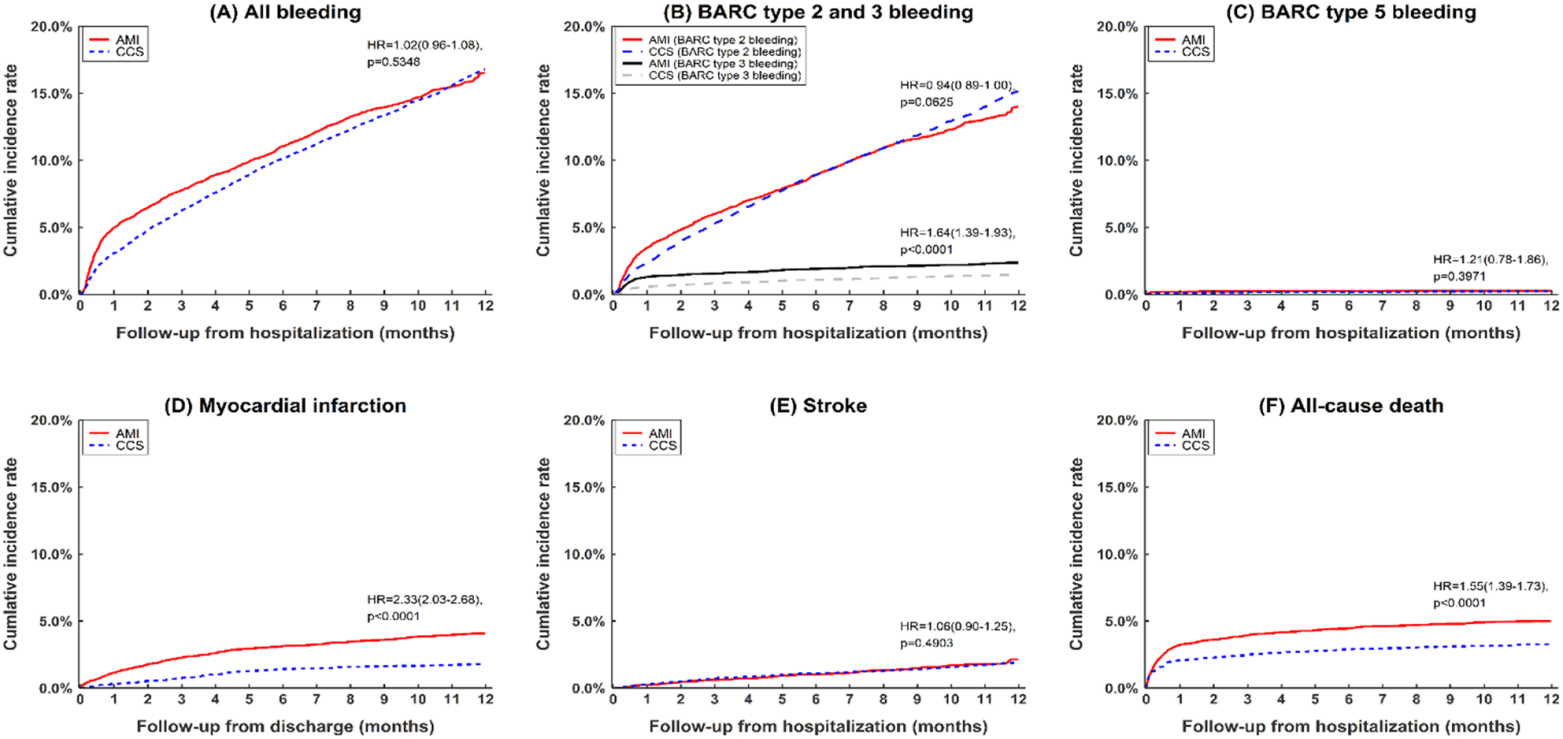
Cumulative incidence rates of clinical outcomes in patients with AMI versus CCS who had received DAPT after coronary stenting (after propensity score weighting). AMI, acute myocardial infarction; BARC, Bleeding Academic Research Consortium; CCS, chronic coronary syndrome.

Figure 2 and Supplemental Table 1 present the 1-year outcomes after propensity score weighting for the AMI and CCS patients who were treated with DAPT after coronary stenting. After weighting, the incidence rate of BARC type 3 bleeding was higher in the AMI patients than in the CCS patients (0.22 vs. 0.13 per 100 person-months; hazard ratio [HR] 1.64; 95% confidence interval [CI] 1.39–1.93; *p* < 0.01), while the incidence rate of BARC type 2 bleeding was numerically in the CCS patients than in the AMI patients, despite the lack of statistical significance (1.32 vs. 1.4 per 100 person-months; HR 0.94; 95% CI 0.89–1.0; *p* = 0.06). The risk of BARC type 5 bleeding was comparable between the two groups at 1 year after the index procedure (0.03 vs. 0.02 per 100 person-months; HR 1.21; 95% CI 0.78–1.86; *p* = 0.4).

The AMI patients had significantly higher incidence rates of all-cause death (0.49 vs. 0.32 per 100 person-months; HR 1.55; 95% CI 1.39–1.73; *p* < 0.01) and postdischarge MI (0.38 vs. 0.16 per 100 person-months; HR 2.33; 95% CI 2.03–2.68; *p* < 0.01) compared with the CCS patients. The incidence rates of overall stroke (0.17 vs. 0.16 per 100 patient-months; HR 1.06; 95% CI 0.9–1.25; *p* = 0.49) and ischemic (0.15 vs. 0.15 per 100 person-months; HR 1.05; 95% CI 0.88–1.25; *p* = 0.6) and hemorrhagic subtypes of stroke (0.01 vs. 0.01 per 100 person-months; HR 0.83; 95% CI 0.45–1.54; *p* = 0.56) were comparable between the two groups.

Supplemental Figure 1 illustrates the temporal changes in the incidence rates of bleeding, MI, stroke, and death in the two groups. Among the patients with AMI, bleeding, death, and recurrent MI occurred most frequently in the first month after the index PCI. The excess bleeding risk in the AMI patients compared with the CCS patients occurred mainly in the first month of DAPT after PCI. Beyond this period, AMI was not associated with a higher risk of bleeding compared with CCS.

### Bleeding sites in AMI and CCS

The temporal changes in the incidence rates of bleeding from various sites were similar to those of overall bleeding and other adverse events (Supplemental Figure 2). The risk of bleeding from each of the sources was highest in the first month after PCI. The majority of bleeding in both groups occurred in the gastrointestinal tract, followed by genitourologic bleeding. The detailed sources of gastrointestinal tract bleeding are illustrated in Supplemental Figure 3.

### Bleeding as an independent predictor of clinical events

To evaluate whether bleeding was an independent predictor of adverse outcomes in patients who received DAPT after PCI, bleeding was added to the Cox proportional hazards model as a time-covariate risk factor (Table 2). After propensity score weighting, bleeding was independently associated with MI, stroke, and all-cause death, with respective HRs of 1.71 (95% CI 1.41–2.06), 2.29 (95% CI 1.95–2.69), and 1.28 (95% CI 1.21–1.36), each with a p value of < 0.01. The results of the PWP model were consistent with Cox regression.

**Table 2:** Hazard ratios for clinical events in patients with AMI versus CCS where bleeding is treated as a time-dependent covariate.

	Cox		Conditional risk set model	
	HR (95% CI)	*p*-value	HR (95% CI)	*p*-value
**Myocardial infarction**				
Before PSW				
Group	2.44 (1.54–3.88)	< 0.001	2.46 (1.55–3.92)	< 0.001
Bleeding	1.79 (1.47–2.18)	< 0.001	1.8 (1.44–2.25)	< 0.001
After PSW				
Group	2.04 (1.34–3.12)	< 0.001	2.08 (1.14–3.78)	0.017
Bleeding	1.71 (1.41–2.06)	< 0.001	1.71 (1.33–2.18)	< 0.001
**Stroke**				
Before PSW				
Group	0.71 (0.47–1.07)	0.099	0.68 (0.45–1.03)	0.072
Bleeding	2.2 (1.87–2.6)	< 0.001	2.18 (1.77–2.69)	< 0.001
After PSW				
Group	1.0 (0.67–1.48)	< 0.998	0.95 (0.59–1.53)	0.83
Bleeding	2.29 (1.95–2.69)	< 0.001	2.26 (1.84–2.77)	< 0.001
**All-cause death**				
Before PSW				
Group	3.89 (3.43–4.4)	< 0.001	3.98 (3.51–4.51)	< 0.001
Bleeding	1.3 (1.23–1.38)	< 0.001	1.34 (1.23–1.45)	< 0.001
After PSW				
Group	1.55 (1.39–1.72)	< 0.001	1.58 (1.32–1.9)	< 0.001
Bleeding	1.28 (1.21–1.36)	< 0.001	1.31 (1.19–1.45)	< 0.001

*AMI* acute myocardial infarction, *CCS* chronic coronary syndrome, *CI* confidence interval, *HR* hazard ratio, *PSW* propensity score weighting.

### Impact of bleeding on subsequent outcomes at 1 year

The characteristics of patients with bleeding in both groups (AMI: n = 2089; CCS: n = 2980) are listed in Supplemental Table 2. After the occurrence of bleeding, 44.7% of the AMI patients and 36.5% of the CCS patients continued DAPT; 48.6% of the AMI patients and 59.4% of the CCS patients received single antiplatelet therapy; 6.7% of the AMI patients and 4.1% of the CCS patients discontinued antiplatelet therapy. Propensity score weighting was performed for the two subgroups to compare subsequent outcomes at 1 year after the index bleeding. Before propensity score weighting, the AMI patients with bleeding had a higher risk of recurrent MI and death and a marginally higher risk of ischemic stroke than the CCS patient with bleeding (Supplemental Table 3). After weighting, bleeding was associated with a higher risk of stroke (0.29 vs. 0.17 per 100 patient-months; HR 1.7; 95% CI 1.17–2.48; *p* = 0.01), particularly ischemic stroke (0.22 vs. 0.13 per 100 patient-months; HR 1.71; 95% CI 1.11–2.63; *p* = 0.02), and a trend toward a higher risk of MI (0.34 vs. 0.25 per 100 patient-months; HR 1.38; 95% CI 0.99–1.91; *p* = 0.06) in the AMI group than in the CCS group (Table 3). All-cause death at 1 year after the index bleeding was comparable between the two groups after propensity score weighting (1.1 vs. 1.04 per 100 patient-month; HR 1.06; 95% CI 0.89–1.26; *p* = 0.52).

**Table 3:** Clinical outcomes at 1 year after bleeding in patients with AMI versus CCS (after propensity score weighting).

	AMI		CCS		Hazard ratio (95% CI)	*p* value
	n	Incidence rate (per 100 person-month)	n	Incidence rate (per 100 person-month)		
**Patients with any bleeding (AMI: n = 1899.01; CCS: n = 2819.12)**						
Myocardial infarction	68.56	0.34 (0.26–0.42)	74.58	0.25(0.19–0.3)	1.38 (0.99–1.91)	0.056
Stroke	57.81	0.29 (0.21–0.36)	51	0.17(0.12–0.21)	1.7 (1.17–2.48)	0.006
Ischemic stroke	43.82	0.22 (0.15–0.28)	38.52	0.13(0.09–0.17)	1.71 (1.11–2.63)	0.015
Hemorrhagic stroke	8.03	0.04 (0.02–0.08)	7.64	0.03(0.01–0.05)	1.57 (0.58–4.23)	0.372
All-cause death	222.8	1.1 (0.95–1.24)	313.87	1.04(0.92–1.15)	1.06 (0.89–1.26)	0.523

*AMI* acute myocardial infarction, *CCS* chronic coronary syndrome, *CI* confidence interval.

## Discussion

This nationwide retrospective cohort study provides the following key findings: (1) AMI was associated with increased risks of MI, all-cause death, and BARC type 3 bleeding at 1 year compared with CCS; (2) bleeding was an independent predictor of MI, stroke, and all-cause death in East Asian patients who received DAPT after coronary stenting; (3) among the patients with bleeding, AMI was associated with a higher risk of ischemic events at 1 year after bleeding, while the mortality risk was comparable between AMI and CCS after propensity score weighting.

Studies on the association between post-PCI bleeding and clinical outcomes have used randomized controlled trials and registry studies of acute coronary syndrome or PCI to compare the risk of death or MI in patients with and without bleeding. However, AMI and CCS are at either end of the spectrum of coronary heart disease, differing in disease acuity, management, and risk profiles of bleeding. We hypothesized that the types, severity, and prognostic relevance of bleeding may differ between AMI and CCS. Recently, both the Academic Research Consortium (ARC) and the Japanese Circulation Society proposed a list of clinical variables to define patients at high bleeding risk (HBR)^24,25^. Compared with the AMI patients, the CCS patients had higher prevalence of the HBR criteria that were applicable in this database study. Although the overall bleeding risk at 1 year was comparable between AMI and CCS, the temporal changes in the incidence rates of BARC type 2 and type 3 differed between the two groups. AMI was associated with a rapid surge in the rates of BARC type 2 and type 3 bleeding in the acute phase, with the cumulative incidence curves separating from those for CCS by 1 month. The use of antithrombotic agents and mechanical circulatory supports as well as clinical factors that were not captured in this study, such as hemodynamic status, white blood cell count, and a potentially higher prevalence of femoral access for urgent PCI, may have contributed to the excessive bleeding risk in the AMI patients in the acute setting^26–28^. While the majority of BARC type 3 bleeding occurred early after the index PCI, the steady increase in BARC type 2 bleeding may reflect chronic bleeding that occurred during maintenance treatment with DAPT. At 1 year, the cumulative incidence rate of BARC type 2 was numerically higher in the CCS patients than in the AMI patients, despite the lack of statistical difference after propensity score weighting. This finding may suggest different bleeding risk profiles of the two groups, as reflected by higher prevalence of the ARC-HBR criteria and other comorbid conditions in the CCS patients than in the AMI patients. The source of bleeding in East Asian patients after PCI has not been thoroughly addressed in the literature. In this study, the majority of bleeding originated from the gastrointestinal tract, followed by genitourologic bleeding. This pattern was similar to the findings of studies performed in Western populations^29,30^. Characterization of the sources and timing of bleeding may facilitate the development of strategies to reduce these complications and thereby improve patient outcomes.

Consistent with previous studies, bleeding was an independent predictor of adverse outcomes in this East Asian populations, regardless of the initial clinical presentation. One explanation for the causal relationship between bleeding and mortality is that the risk factors of bleeding often overlap with the predictors of ischemic events^12^. Bleeding acting as an indicator of increased ischemic risk may therefore contribute to subsequent mortality. Other possible mechanisms include hemodynamic effect of severe hemorrhage, intracranial bleeding with massive effect, disparity between oxygen supply and demand, blood transfusion, and discontinuation of antiplatelet therapy^12,31^. Scant data exist regarding the comparative outcomes after bleeding in patients with AMI versus CCS. In this study, bleeding confers a greater hazard for subsequent ischemic events in the AMI patients than in the CCS patients. It is conceivable that myocardium already compromised by acute infarction may be particularly vulnerable to imbalanced oxygen supply and demand following bleeding complications. Furthermore, AMI is associated with a heightened state of inflammation, platelet activation and thrombosis, compared with a chronic inflammatory response in patients with CCS^32–34^. Withholding antiplatelet therapy after bleeding may result in activation of platelets and the coagulation cascade, leading to a higher risk of stent thrombosis and other ischemic events in patients with AMI versus CCS^35,36^. Ischemic stroke is a rare but serious complication after AMI, with a decreasing incidence over the past years due to the use of aspirin, P2Y_12_ inhibitors, and statins^37,38^. The leading cause of ischemic stroke following AMI is cardiac embolism, caused by mural thrombus in akinetic segments of the left ventricle or due to new onset atrial fibrillation^38^. The incidence of stroke after MI is highest in the acute phase but remains elevated thereafter^39^. Therefore, discontinuation of antiplatelet therapy to minimize bleeding may confer a higher risk of ischemic stroke in patients with AMI than in those with CCS.

Previous studies have revealed a temporal relationship between bleeding and mortality^7,40^ and an increased hazard of morality with higher BARC grades^41,42^. In this study, bleeding occurred earlier and often in a more severe form (BARC type 3) in the AMI patients than in the CCS patients. Therefore, 1-year mortality after bleeding was higher in the AMI patients than in the CCS patients before propensity score weighting. After adjusting for the clinical variables, however, there was no significant difference in the 1-year mortality after bleeding between the two groups. AMI and CCS differ remarkably with respect to underlying pathophysiology, disease acuity, and inhospital management. Antithrombotic therapy is the cornerstone of pharmacologic management in AMI. The use of antithrombotic agents, particularly glycoprotein IIb/IIIa inhibitors (26.2% vs. 3.0%), and intra-aortic balloon pump (9.0% vs. 1.1%) were much higher in the AMI patients than in the CCS patients. Both antithrombotic therapy and mechanical circulatory supports have been documented to increase major bleeding in patients with acute coronary syndrome^43,44^. Therefore, balancing the difference in these variables between the two groups may have also attenuated the difference in the prognostic impact of bleeding on patients with AMI versus CCS.

This retrospective database analysis has several inherent limitations. First, Taiwan’s NHIRD does not provide data on vital signs, laboratory tests, echocardiographic parameters, and procedural or angiographic details of PCI. Not all the ARC-HBR criteria were applicable in our analysis. Due to the lack of causes of death, we could not determine the mechanistic link between bleeding and mortality, nor could we investigate how the unmeasured variables may have affected the results. Second, this study aimed to evaluate the clinical impact of bleeding during DAPT. To eliminate confounding caused by different antithrombotic regimens, we excluded the patients who received oral anticoagulation or those who did not receive DAPT with aspirin and clopidogrel. The study period predates the introduction of ticagrelor and prasugrel in Taiwan. The efficacy and safety of these novel P2Y_12_ inhibitors in East Asian patients remain to be investigated. Third, the ICD-9-CM codes do not contain the diagnosis of vascular access site bleeding. The incidence rate and the clinical impact of access site bleeding could not be estimated in our study. Forth, unlike MI, stroke, and death, bleeding has not been validated in the NHIRD of Taiwan. However, insurance claims for each health care encounter were all reviewed and inspected by medical reimbursement specialists. The possibility of incorrect coding should be low. To avoid repeat calculations, we excluded the patients with previous bleeding at enrollment and only the first bleeding was analyzed during follow-up. Therefore, the real incidence of bleeding may be underestimated in our study cohort. Finally, our results may not be generalized to patients who receive CABG for AMI or CCS nor to Western populations.

In conclusion, bleeding was an independent predictor of MI, stroke, and death in East Asian patients who were on DAPT after PCI. AMI was associated with an increased risk of BARC type 3 bleeding compared with CCS. While bleeding conferred a higher risk of ischemic events in patients with AMI than in patients with CCS, 1-year mortality after bleeding was comparable between the two groups.

## Supplementary information

Supplementary Figure 1.

Supplementary Figure 2.

Supplementary Figure 3.

Supplementary Tables.

Supplementary Figure Legends.
