# Supplementary figures and images for: Impact of bleeding during dual antiplatelet therapy in patients with coronary artery disease
Source: Sci Rep. 2020 Dec 7;10:21345. doi: 10.1038/s41598-020-78400-4 (PMC7721794; doi:10.1038/s41598-020-78400-4)

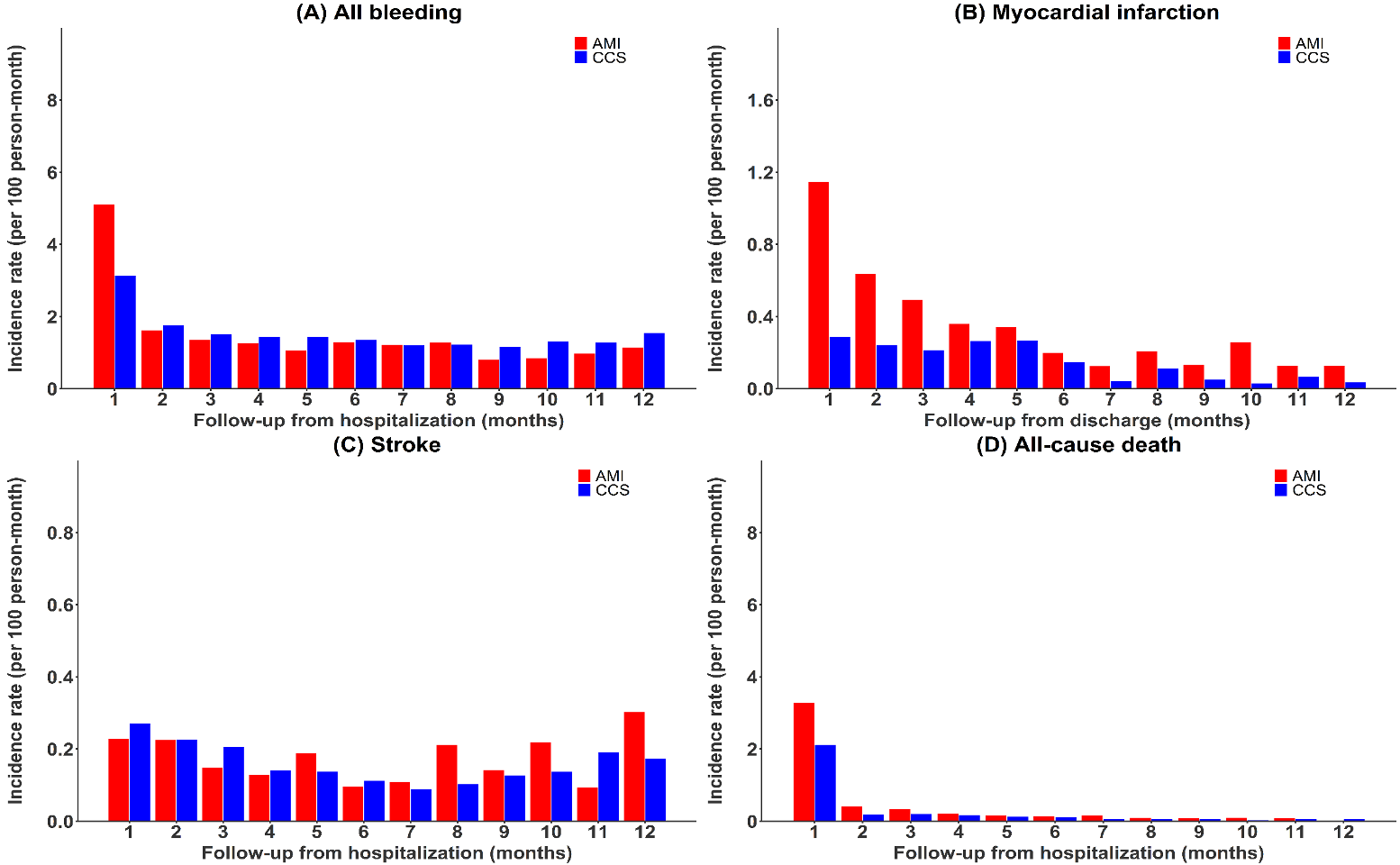

Supplement: Supplementary file 1 — Supplementary Figure 1. [file 41598_2020_78400_MOESM1_ESM.png]

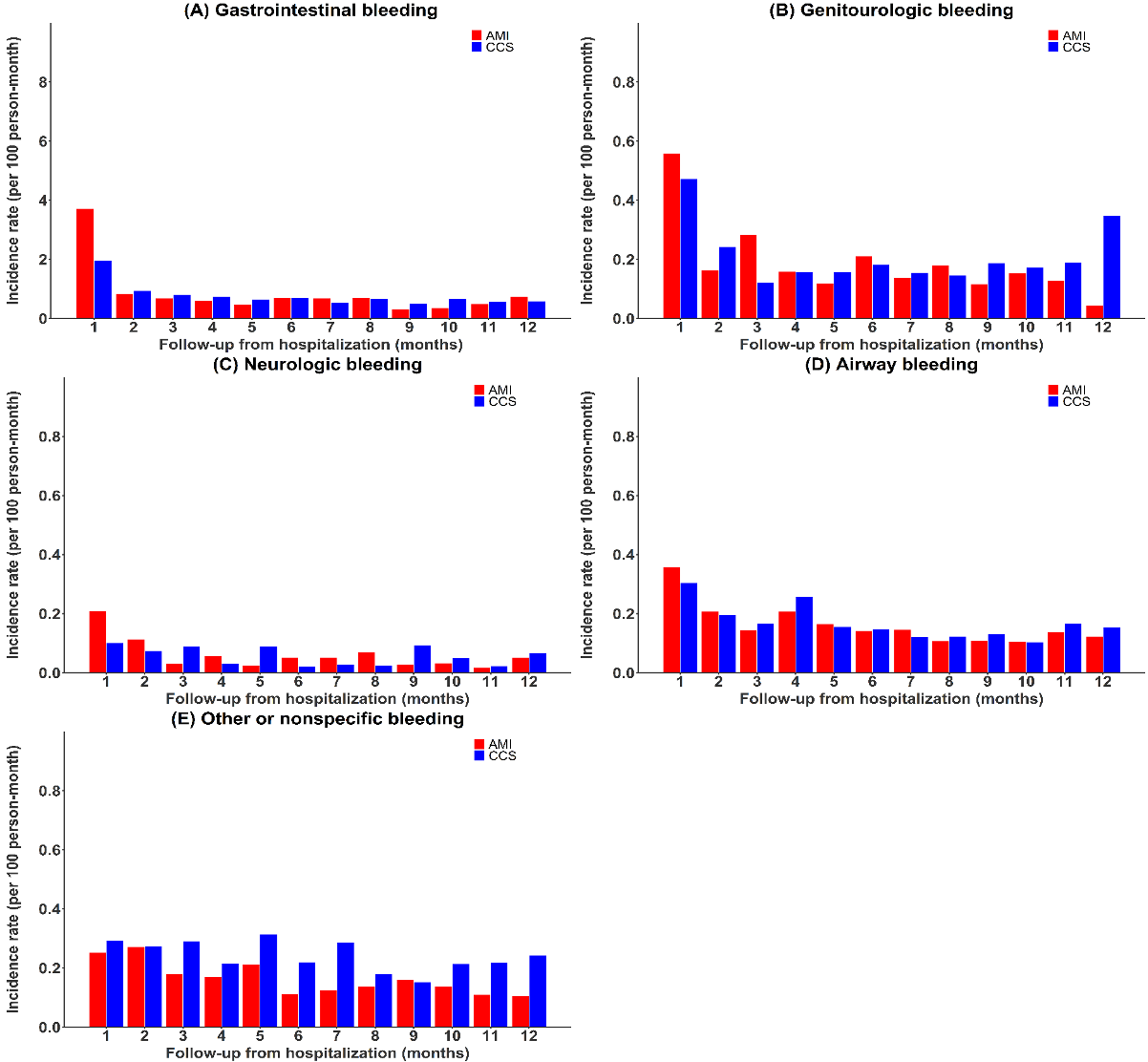

Supplement: Supplementary file 2 — Supplementary Figure 2. [file 41598_2020_78400_MOESM2_ESM.png]

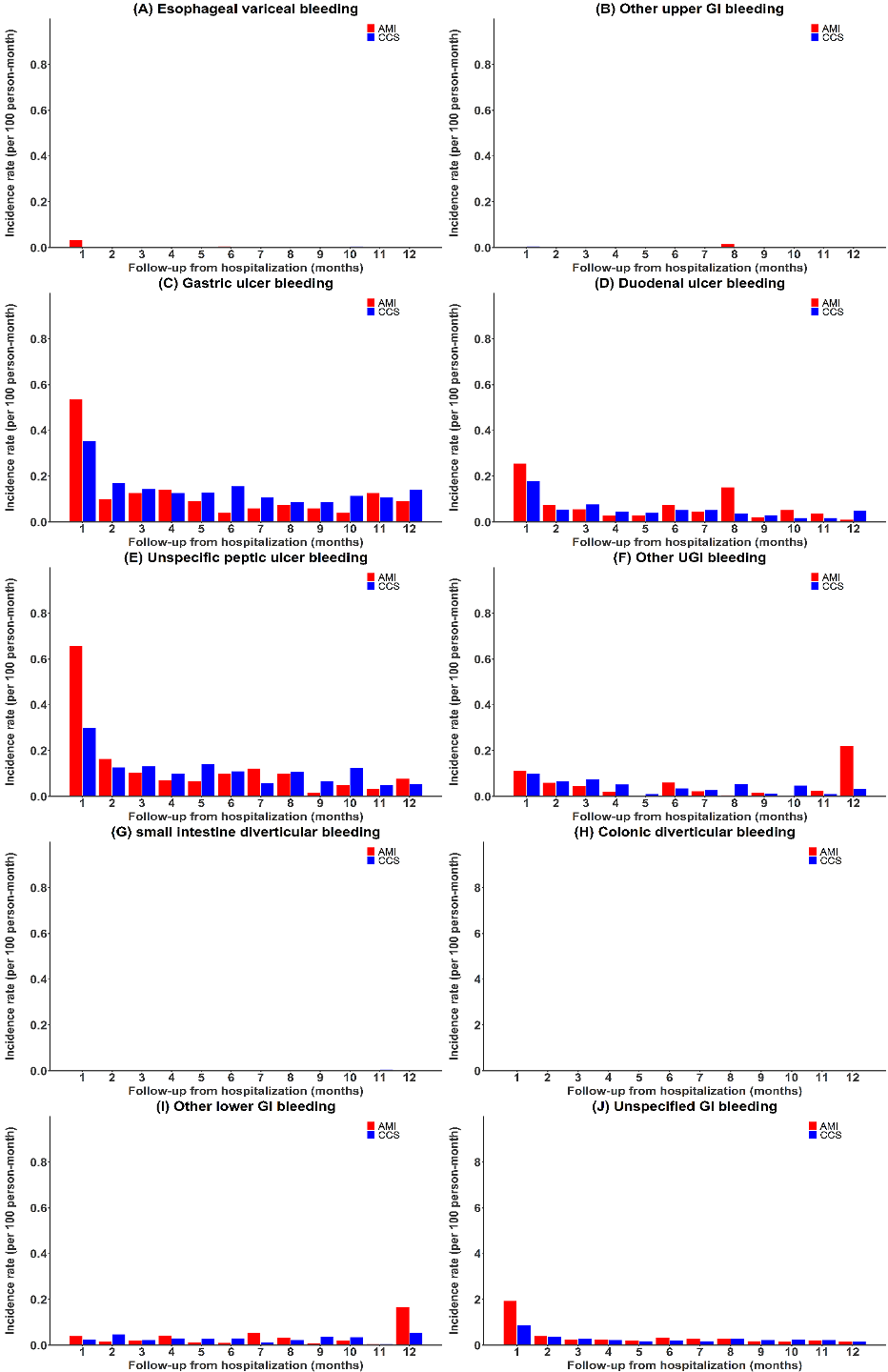

Supplement: Supplementary file 3 — Supplementary Figure 3. [file 41598_2020_78400_MOESM3_ESM.png]
