# Supplementary material for: Impact of bleeding during dual antiplatelet therapy in patients with coronary artery disease
Source: Sci Rep. 2020 Dec 7;10:21345. doi: 10.1038/s41598-020-78400-4 (PMC7721794; doi:10.1038/s41598-020-78400-4)
Supplement: Supplementary file 4 — Supplementary Tables. [file 41598_2020_78400_MOESM4_ESM.docx]

**Impact of Bleeding During Dual Antiplatelet Therapy in Patients with Coronary Artery Disease**

Brief title: Impact of bleeding during DAPT

Ying-Chang Tung,^1,2^ Lai-Chu See,^3,4,5^ Shu-Hao Chang,^3^ Jia-Rou Liu,^3^ Chi-Tai Kuo,^1,2^ Chi-Jen Chang^1,2*^

From the ^1^Cardiovascular Department, Linkou Chang Gung Memorial Hospital, Taoyuan, Taiwan; ^2^College of Medicine, Chang Gung University, Taoyuan, Taiwan;

^3^Department of Public Health, College of Medicine, Chang Gung University, Taoyuan, Taiwan; ^4^Biostatistics Core Laboratory, Molecular Medicine Research Center, Chang Gung University, Taoyuan, Taiwan; and ^5^Division of Rheumatology, Allergy and Immunology, Department of Internal Medicine, Linkou Chang Gung Memorial Hospital, Taoyuan, Taiwan.

The authors take responsibility for all aspects of the reliability and freedom from bias of the data presented and their discussed interpretation

*Correspondence:

Chi-Jen Chang, MD

Cardiovascular Department, Linkou Chang Gung Memorial Hospital

No. 5, Fusing St., Gueishan Dist., Taoyuan City 33305, Taiwan (R.O.C.)

E-mail: chijenformosa@gmail.com

Phone: + (886)-3-328-1200 ext. 8162

Fax: + (886)-3-327-1192

**Supplemental Table 1. Clinical outcomes at 1 year in patients with AMI vs. CCS who received DAPT after coronary stenting**

|  |  | Before PSW | | | | |  | |  | |  | | |  | | | After PSW | | | | | | |  | | | |  | | | |  |  |
| --- | --- | --- | --- | --- | --- | --- | --- | --- | --- | --- | --- | --- | --- | --- | --- | --- | --- | --- | --- | --- | --- | --- | --- | --- | --- | --- | --- | --- | --- | --- | --- | --- | --- |
|  | AMI (n=15391) | | | CCS (n=19724) | | | |  | |  | |  | | | AMI (n=14840.13) | | | | | CCS (n=19534.12) | | | | |  | | | |  | | | |  |
|  | n | Incidence rate^c^ (95% CI) | n | | Incidence rate^c^ (95% CI) | | Hazard ratio (95% CI) | | p value | |  | | | n | | | Incidence rate^c^ (95% CI) | | n | | | Incidence rate^c^ (95% CI) | | Hazard ratio (95% CI) | | | p value | | | |  |  |  |
| All bleeding ^a^ | 2089 | 1.52 (1.45-1.58) | 2980 | | 1.64 (1.58-1.7) | | 0.93 (0.88-0.98) | | 0.01 | |  | | | 2158.9 | | | 1.61 (1.54-1.68) | | 2817.3 | | | 1.58 (1.52-1.64) | | 1.02 (0.96-1.08) | | | 0.535 | | | |  |  |  |
| BARC type 2 bleeding^a^ | 1710 | 1.22 (1.17-1.28) | 2699 | | 1.47 (1.42-1.53) | | 0.83 (0.78-0.88) | | <0.001 | |  | | | 1800.2 | | | 1.32 (1.26-1.38) | | 2516.5 | | | 1.4 (1.35-1.46) | | 0.94 (0.89-1) | | | 0.063 | | | |  |  |  |
| BARC type 3 bleeding^a^ | 320 | 0.22 (0.19-0.24) | 250 | | 0.13 (0.11-0.14) | | 1.7 (1.44-2.01) | | <0.001 | |  | | | 319.45 | | | 0.22 (0.2-0.24) | | 258.01 | | | 0.13 (0.12-0.15) | | 1.64 (1.39-1.93) | | | <0.001 | | | |  |  |  |
| BARC type 5 bleeding^a^ | 59 | 0.04 (0.03-0.05) | 31 | | 0.02 (0.01-0.02) | | 2.49 (1.62-3.85) | | <0.001 | |  | | | 39.19 | | | 0.03 (0.02-0.03) | | 42.75 | | | 0.02 (0.02-0.03) | | 1.21 (0.78-1.86) | | | 0.397 | | | |  |  |  |
| Myocardial infarction^b^ | 558 | 0.39 (0.36-0.42) | 260 | | 0.13 (0.12-0.15) | | 2.9 (2.5-3.36) | | <0.001 | |  | | | 539.54 | | | 0.38 (0.35-0.42) | | 308.81 | | | 0.16 (0.14-0.18) | | 2.33 (2.03-2.68) | | | <0.001 | | | |  |  |  |
| Stroke ^a^ | 211 | 0.14 (0.12-0.16) | 333 | | 0.17 (0.15-0.19) | | 0.84 (0.7-0.99) | | 0.041 | |  | | | 249.45 | | | 0.17 (0.15-0.19) | | 309.2 | | | 0.16 (0.14-0.18) | | 1.06 (0.9-1.25) | | | 0.49 | | | |  |  |  |
| Ischemic stroke^a^ | 193 | 0.13 (0.11-0.15) | 305 | | 0.15 (0.14-0.17) | | 0.83 (0.7-1) | | 0.049 | |  | | | 223.42 | | | 0.15 (0.13-0.17) | | 280.04 | | | 0.15 (0.13-0.16) | | 1.05 (0.88-1.25) | | | 0.598 | | | |  |  |  |
| Hemorrhagic stroke^a^ | 15 | 0.01 (0-0.02) | 25 | | 0.01 (0.01-0.02) | | 0.79 (0.42-1.51) | | 0.48 | |  | | | 16.52 | | | 0.01 (0.01-0.02) | | 26.12 | | | 0.01 (0.01-0.02) | | 0.83 (0.45-1.54) | | | 0.558 | | | |  |  |  |
| All-cause death ^a^ | 989 | 0.66 (0.62-0.7) | 337 | | 0.17 (0.15-0.19) | | 3.86 (3.42-4.37) | | <0.001 | |  | | | 720.11 | | | 0.49 (0.45-0.52) | | 610.56 | | | 0.32 (0.29-0.34) | | 1.55 (1.39-1.73) | | | <0.001 | | | |  |  |  |
| ***^a^During hospitalization and after discharge*** | | | |  | |  | |  | |  | | |  | | |  | |  | | |  | |  | | |  | | | |  | | | |
| ***^b^After discharge*** |  |  |  | |  | |  | |  | |  | | |  | | |  | |  | | |  | |  | | | |  | | | |  |  |
| ***^c^Per 100 person-months*** | | | |  | |  | |  | |  | | |  | | |  | |  | | |  | |  | | |  | | | |  | | | |

Abbreviations:

AMI, acute myocardial infarction; BARC, Bleeding Academic Research Consortium; CCS, chronic coronary syndrome; CI, confidence interval; DAPT, dual antiplatelet therapy; PSW, propensity score weighting.

**Supplemental Table 2. Demographics and clinical characteristics of patients with bleeding during DAPT**

|  | Before propensity score weighting | | |  | After propensity score weighting | | |
| --- | --- | --- | --- | --- | --- | --- | --- |
|  | AMI (n=2089) | CCS (n=2980) | SMD |  | AMI (n=1913.52) | CCS (n=2831.64) | SMD |
|  | n (%) | n (%) |  |  | n (%) | n (%) |  |
| Age, years | 66.31 (12.89) | 67.73 (11.28) | -0.117 |  | 66.91 (12.17) | 67.43 (11.46) | -0.044 |
| ≤75 | 1490 (71.33%) | 2119 (71.11%) | -0.005 |  | 1343.13 (70.19%) | 2011.19 (71.03%) | 0.018 |
| >75 | 599 (28.67%) | 861 (28.89%) |  |  | 570.39 (29.81%) | 820.45 (28.97%) |  |
| Sex |  |  | 0.123 |  |  |  | 0.037 |
| Female | 486 (23.26%) | 853 (28.62%) |  |  | 478.37 (25%) | 754.24 (26.64%) |  |
| Male | 1603 (76.74%) | 2127 (71.38%) |  |  | 1435.14 (75%) | 2077.40 (73.36%) |  |
| **Comorbidities** |  |  |  |  |  |  |  |
| Diabetes mellitus | 889 (42.56%) | 1469 (49.3%) | -0.136 |  | 906.59 (47.38%) | 1341.16 (47.36%) | <0.001 |
| Hypertension | 1615 (77.31%) | 2594 (87.05%) | -0.257 |  | 1578.95 (82.52%) | 2382.18 (84.13%) | -0.043 |
| Atrial fibrillation | 101 (4.83%) | 188 (6.31%) | -0.064 |  | 102.25 (5.34%) | 167.46 (5.91%) | -0.025 |
| Congestive heart failure | 590 (28.24%) | 794 (26.64%) | 0.036 |  | 537.21 (28.07%) | 753.29 (26.6%) | 0.033 |
| Chronic kidney disease | 436 (20.87%) | 854 (28.66%) | -0.181 |  | 473.57 (24.75%) | 739.65 (26.12%) | -0.032 |
| Chronic obstructive pulmonary disease | 446 (21.35%) | 743 (24.93%) | -0.085 |  | 436.85 (22.83%) | 673.28 (23.78%) | -0.022 |
| Chronic liver disease | 239 (11.44%) | 486 (16.31%) | -0.141 |  | 254.20 (13.28%) | 409.85 (14.47%) | -0.034 |
| Previous stroke | 192 (9.19%) | 377 (12.65%) | -0.111 |  | 213.73 (11.17%) | 328.90 (11.62%) | -0.014 |
| Hyperlipidemia | 1602 (76.69%) | 2234 (74.97%) | 0.040 |  | 1449.10 (75.73%) | 2156.72 (76.17%) | -0.01 |
| End stage renal disease | 125 (5.98%) | 336 (11.28%) | -0.189 |  | 164.87 (8.62%) | 265.52 (9.38%) | -0.027 |
| Anemia | 282 (13.5%) | 523 (17.55%) | -0.112 |  | 293.52 (15.34%) | 455.56 (16.09%) | -0.021 |
| Thrombocytopenia | 7 (0.34%) | 16 (0.54%) | -0.031 |  | 5.63 (0.29%) | 13.48 (0.48%) | -0.029 |
| **Medications** |  |  |  |  |  |  |  |
| ACE inhibitor | 1668 (79.85%) | 1623 (54.46%) | 0.561 |  | 1289.41 (67.38%) | 1814.59 (64.08%) | 0.07 |
| ARB | 1242 (59.45%) | 1855 (62.25%) | -0.057 |  | 1178.23 (61.57%) | 1733.76 (61.23%) | 0.007 |
| Beta-blocker | 1763 (84.39%) | 2494 (83.69%) | 0.019 |  | 1610.50 (84.16%) | 2362.17 (83.42%) | 0.02 |
| Statin | 1558 (74.58%) | 2077 (69.7%) | 0.109 |  | 1393.65 (72.83%) | 2035.55 (71.89%) | 0.021 |
| PPI | 833 (39.88%) | 1190 (39.93%) | -0.001 |  | 787.60 (41.16%) | 1112.44 (39.29%) | 0.038 |
| H2 blocker | 398 (19.05%) | 563 (18.89%) | 0.004 |  | 355.10 (18.56%) | 528.69 (18.67%) | -0.003 |
| Steroid | 105 (5.03%) | 155 (5.2%) | -0.008 |  | 100.33 (5.24%) | 149.40 (5.28%) | -0.002 |
| NSAID | 348 (16.66%) | 486 (16.31%) | 0.009 |  | 312.38 (16.32%) | 456.23 (16.11%) | 0.006 |
| **Inhospital management** |  |  |  |  |  |  |  |
| Unfractionated heparin | 1706 (81.67%) | 1961 (65.81%) | 0.366 |  | 1433.14 (74.9%) | 2029.98 (71.69%) | 0.073 |
| Enoxaparin | 673 (32.22%) | 771 (25.87%) | 0.140 |  | 565.57 (29.56%) | 792.11 (27.97%) | 0.035 |
| GP IIb/IIIa inhibitor | 547 (26.18%) | 89 (2.99%) | 0.696 |  | 263.12 (13.75%) | 305.88 (10.8%) | 0.09 |
| IABP | 187 (8.95%) | 32 (1.07%) | 0.367 |  | 90.92 (4.75%) | 96.86 (3.42%) | 0.067 |
| **DAPT after bleeding occurred** |  |  | 0.285 |  |  |  | 0.046 |
| None | 140 (6.7%) | 123 (4.13%) |  |  | 103.97 (5.43%) | 146.04 (5.16%) |  |
| Aspirin alone | 422 (20.2%) | 934 (31.34%) |  |  | 486.67 (25.43%) | 768.85 (27.15%) |  |
| Clopidogrel alone | 593 (28.39%) | 835 (28.02%) |  |  | 536.98 (28.06%) | 777.96 (27.47%) |  |
| DAPT continued | 934 (44.71%) | 1088 (36.51%) |  |  | 785.89 (41.07%) | 1138.80 (40.22%) |  |

Abbreviations:

ACE, angiotensin-converting enzyme; AMI, acute myocardial infarction; ARB, angiotensin II receptor blocker; CCS, chronic coronary syndrome; DAPT, dual antiplatelet therapy; GP, glycoprotein; IABP, intra-aortic balloon pump; NSAID, nonsteroidal anti-inflammatory drug; PPI, proton-pump inhibitor; SMD, standardized mean difference.

**Supplemental Table 3. Clinical outcomes at 1 year after bleeding in patients with AMI vs. CCS (before propensity score weighting)**

|  |  | AMI | |  | CCS | | |  | |  | |
| --- | --- | --- | --- | --- | --- | --- | --- | --- | --- | --- | --- |
|  |  | n | Incidence rate (per 100 person-months) |  | n | Incidence rate (per 100 person-months) | Hazard ratio (95% CI) | | p value | |  |
| Patients with any bleeding | Myocardial infarction | 73 | 0.34 (0.26-0.42) |  | 62 | 0.19 (0.15-0.24) | 1.75 (1.25-2.45) | | 0.001 | |  |
| (AMI: n=2089; CCS: n=2980) | Stroke | 53 | 0.25 (0.18-0.31) |  | 56 | 0.17 (0.13-0.22) | 1.4 (0.96-2.04) | | 0.078 | |  |
|  | Ischemic stroke | 42 | 0.19 (0.13-0.25) |  | 43 | 0.13 (0.09-0.17) | 1.45 (0.95-2.21) | | 0.089 | |  |
|  | Hemorrhagic stroke | 7 | 0.03 (0.01-0.07) |  | 9 | 0.03 (0.01-0.05) | 1.14 (0.42-3.06) | | 0.794 | |  |
|  | All-cause death | 283 | 1.3 (1.15-1.45) |  | 294 | 0.91 (0.81-1.02) | 1.41 (1.2-1.66) | | <0.001 | |  |

Abbreviations: AMI, acute myocardial infarction; CCS, chronic coronary syndrome; CI, confidence interval.
