# Supplementary material for: Impact of bleeding during dual antiplatelet therapy in patients with coronary artery disease
Source: Sci Rep. 2020 Dec 7;10:21345. doi: 10.1038/s41598-020-78400-4 (PMC7721794; doi:10.1038/s41598-020-78400-4)
Supplement: Supplementary file 5 — Supplementary Figure Legends. [file 41598_2020_78400_MOESM5_ESM.docx]

**Supplemental Figure 1. Incidence rates of clinical outcomes per month in patients with AMI vs. CCS who had received DAPT after coronary stenting.**

AMI, acute myocardial infarction; CCS, chronic coronary syndrome.

**Supplemental Figure 2. Incidence rates of bleeding per month according to bleeding sites.**

AMI, acute myocardial infarction; CCS, chronic coronary syndrome.

**Supplemental Figure 3. Sources of gastrointestinal tract bleeding in patients with AMI vs. CCS.**

AMI, acute myocardial infarction; CCS, chronic coronary syndrome.
